# The Probiotication of a Lychee Beverage with *Saccharomyces boulardii*: An Alternative to Dairy-Based Probiotic Products

**DOI:** 10.3390/foods14020156

**Published:** 2025-01-07

**Authors:** Marcela Moreira Terhaag, Otávio Akira Sakai, Fabiana Ruiz, Sandra Garcia, Fernando Rodrigo Bertusso, Sandra Helena Prudêncio

**Affiliations:** 1 Federal Institute of Paraná (IFPR), Umuarama 87507-014, Brazil; otavio.sakai@ifpr.edu.br (O.A.S.); fabi_ruiz17@hotmail.com (F.R.); 2 Department of Food Science and Technology, Londrina State University, Londrina 86051-970, Brazil; gassandra15@gmail.com (S.G.); sandrah@uel.br (S.H.P.); 3 State Secretariat of Education of Paraná, UNIALFA-Umuarama, Umuarama 87502-000, Brazil; bertusso@gmail.com

**Keywords:** juice, fermentation, yeast, storage, functional, *Litchi chinensis*

## Abstract

Probiotic vegetable-based beverages, such as lychee, can be rich in nutrients, free of cholesterol and lactose, and also have few allergenic components. *Saccharomyces boulardii* is an alternative to make lychee juice a probiotic beverage. This work aimed to develop probiotic lychee beverage (LB) using *S. boulardii* by evaluating the effect of refrigerated storage on cell viability, physicochemical characteristics, and acceptance. LB supplemented with *S. boulardii* was fermented (24 h/30 °C), supplemented with sucrose (4–12 °Brix), and refrigerated (up to 28 days/4 °C). The yeast viability, total soluble solid (TSS) and, ethanol content, pH, total phenolic compounds (TPC), and antioxidant activity (AA) levels were evaluated over 28 days of storage. Also, the profiles of sugars, organic acids, and phenolic were determined via chromatographic analysis. The sensory acceptance of the probiotic beverage was evaluated. Higher sucrose levels (12 °Brix) resulted in greater yeast viability (6.9 log CFU/mL) on the 21st day of storage and superior TPC (153 µmol TEAC/mL) and ethanol levels (8.7%). Storage reduced the TPC, AA, and TSS. LB supplemented with sucrose to 12 °Brix, probioticated by *S. boulardii*, and stored for 21 days became accepted by the consumer and presented an adequate physicochemical profile with probiotic potential. The probiotication of lychee beverage is an alternative to dairy-based probiotic beverages.

## 1. Introduction

Probiotics are non-pathogenic microorganisms that, when administered in adequate amounts, exert a positive influence on the health or physiology of the host [1,2]. Microorganisms with probiotic effects include bacteria of the genera *Lactobacillus* and *Bifidobacterium* in addition to the yeast *Saccharomyces boulardii* [3].

*S. boulardii* is a non-pathogenic thermophilic yeast that is resistant to gastric acid and proteolysis and was isolated from lychee in Indochina [3,4,5]. It is not part of the intestinal microbiota; it does not colonize the intestine, and its concentration decreases after the end of administration [4,6]. This yeast has been used to treat traveler’s diarrhea, antibiotic-associated diarrhea, acute gastroenteritis in adults and children, diarrhea in intensive care unit patients, metabolic syndrome treatment, and chronic diarrhea in HIV-positive patients [4,7,8,9]. It has a protective effect against *Clostridium difficile*, *Vibrio cholerae*, *Shigella* and *E. coli* (EPEC), and *Salmonella enterica* Typhimurium [4,10,11].

Probiotication is the process of inoculating beneficial microorganisms into a liquid substrate to produce functional beverages, which subsequently adds market value due to the various health benefits of probiotics [12,13]. Fruit and vegetable juices are appropriate as a good matrix for probiotics because they do not contain dairy allergens that prevent consumption by the population [13,14].

Despite providing products with probiotic potential and being sensorially accepted, the use of *S. boulardii* in beverages has not been commercially explored [14,15]. Several authors have noted the possibility of applying *S. boulardii* in different food matrices, such as tomato and carrot juice [14]; craft beer [10,16]; rose wine [17]; grape juice with apple pieces [18]; beverages made from radish, beetroot; and carrot [14]; apple pulp [19]; beetroot, carrot, and celery beverages [20]; tomato juice [21]; milk and dairy drinks [22]; goat milk yogurt [23]; berry juice [5]; and soymilk ice cream [24], among others [25].

One of the challenges of using *S. boulardii* as a probiotic in food is that it produces ethanol as a metabolic product in addition to CO_2_. Considering that the maximum permitted ethanol content in a beverage labeled as probiotic varies according to the country and specific regulations [26,27], the presence of ethanol is not an impediment to the functionality of a beverage or to the use of *S. boulardii* in food, since it remains viable in these beverages [28,29]. Queipo-Ortuño et al. [30] report that even for red wine, there are select gut microbiota in humans, possibly due to red wine polyphenols. Thus, as long as probiotication is associated with a product with an adequate profile of polyphenols or other bioactive, ethanol may not impede the development of some probiotic microorganisms.

Lychee (*Litchi chinensis* Sonn.), which was originally obtained from China [31,32], is a sweet fruit that is rich in sugars; has a fleshy pulp, an attractive color, and aroma; and can be consumed in syrup, powdered pulp, dehydrated, and in the form of juice [33]. This fruit is rich in bioactive components such as gallic acid, chlorogenic acid, (+)-catechin, caffeic acid, (−)-epicatechin, rutin, quercetin 3-rut-7-rha, and epicatechin [31,32,33].

In addition to presenting bioactive compounds with antioxidant, antibacterial, anti-inflammatory, antiallergic, hepatoprotective, antiviral, anticancer, and vasodilator properties [32,33,34], lychee has a rich and pleasant flavor, with good sensory characteristics and potential application in fermented and probiotic beverages [32,33,34,35].

Thus, the objective of this study was to develop a probiotic lychee beverage using the yeast *Saccharomyces boulardii* and to evaluate the effect of refrigerated storage on cell viability, physicochemical characteristics, and sensory acceptance.

## 2. Materials and Methods

### 2.1. Material

#### 2.1.1. Reagents

The following reagents from Sigma–Aldrich (St. Louis, MI, USA) (purity ≥ 99%) were used: glucose, fructose, sucrose, stachyose, mannitol, citric acid, malic acid, tartaric acid, lactic acid, succinic acid, acetic acid, ascorbic acid, DPPH, TPTZ (2,4,6-tripyridyl-S-triazine), ABTS, Folin-Ciocalteu, Trolox, gallic acid, protocatechuic acid, theobromine, paraxanthin, epigallocatechin, catechin, epicatechin, quercetin, caffeine, caffeine, rutin, kaempferol, chlorogenic acid, p-coumaric acid, ferrulic acid, synapic acid, myricetin, and trigonelline. Acetonitrile (HPLC-grade) was obtained from J.T. Baker (Avantor, Radnor, Pennsylvania). Ethanol and other reagents were obtained from Merck (Darmstadt, Germany). Ultrapure water (Simplicity 185, Millipore, MA, USA) was used for the extractions and standard solutions.

#### 2.1.2. Lychee Juice

Lychees of the Bengal cultivar (23°45′57′′ S; 53°19′30′′ O; altitude of 442 m) were sanitized (NaClO 0.01% per 5 min), peeled, and pulped (F. Silva, model MS-200, Joao Pessoa, Brazil). The pulp (15.8 °Brix) was packed in polyethylene bags, stored at −15 °C, and filtered through 200 mesh, then gently pressed by hand to obtain the lychee juice.

#### 2.1.3. Probiotic Culture

*S. boulardii* CNCM I-745 was obtained from a commercial lyophilized product (Floratil^®^ Merck S.A., São Paulo, Brazil). Each 100 mg capsule contained at least 8.66 log *S. boulardii*-17 cells.

### 2.2. Methods

#### 2.2.1. Preparation of *S. boulardii* Inoculum

The lyophilized yeast was reactivated in 100 mL of YPD broth (1:2:2:95, yeast extract, bacteriological peptone, dextrose, and water) and stirred (120 rpm) at 30 °C for 24 h (CT-712 Cientec, Porto Alegre, Brazil). After incubation, the yeast biomass was centrifuged at 14,000× *g* for 15 min (Eppendorf 5804 R, Hamburg, Germany) and washed twice with NaCl 0.85% (*w*/*v*) to remove residual YPD. The biomass was resuspended in 50 mL of 0.85% NaCl (*w*/*v*), resulting in the activated *S. boulardii* culture.

#### 2.2.2. Probiotication of Lychee Juice

**Figure 1:**
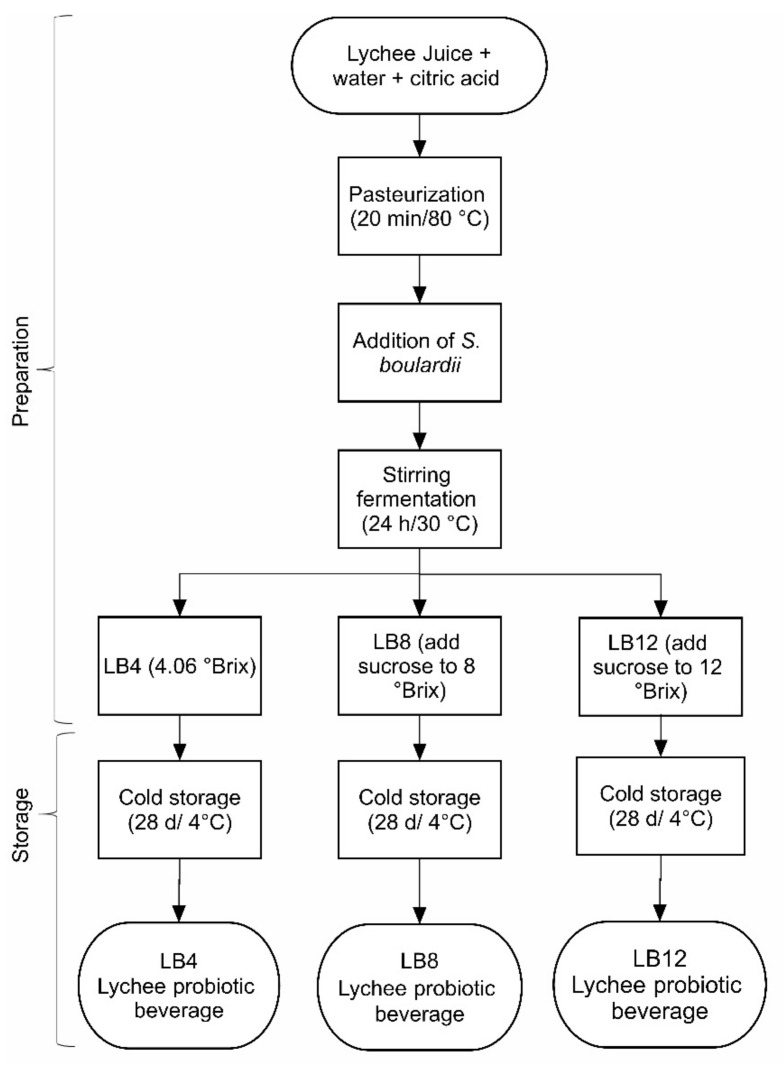
Main steps of probiotication of lychee juice.

Figure 1 shows the main stages of the probiotication of lychee juice, as proposed from preliminary tests. Water and lychee juice were combined until a total soluble solid (TSS) concentration of 12 °Brix was reached. The pH was adjusted to 4.5, and 20% citric acid was added to the juice. The juices were glass bottled and pasteurized in a water bath (20 min/80 °C).

Lychee juice was inoculated with *S. boulardii* to reach 5 log cells/mL of juice and fermented in an oven with agitation (120 rpm) at 24 h/30 °C. The probioticed juice (LB) (4.07 °Brix) was separated into three portions: (a) the first remaining with the original TSS content (~4 °Brix), named LB4; (b) another receiving sucrose to reach 8 °Brix, denominated LB8; and the last portion (c) receiving the addition of sucrose up to 12 °Brix, denominated LB12. These beverages were stored in a refrigerator at 4 °C for 28 days and analyzed every seven days. All the juices were prepared twice.

#### 2.2.3. Viability of *S. boulardii*

The viability of *S. boulardii* was determined after dilution of the beverages in 0.1% (*w*/*v*) peptone water (Oxoid^®^), followed by plating on YPD agar at 37 °C for 48 h. The results are expressed as log CFU/mL.

#### 2.2.4. Physicochemical Analysis

The pH was determined using a pH meter (model HI 3221; Hanna Instruments Inc., Woonsocket, RI, USA). The content of total soluble solids (TSSs) in °Brix was determined using a digital refractometer (Pocket Pal-1, Atago, Tokyo, Japan).

The total phenolic compound (TPC) content was determined according to the methods of Singleton, Orthofer, and Lamuela-Raventos [36], and the results are expressed as µg of gallic acid equivalents (GAE) per mL.

Antioxidant activity (AA) was evaluated by measuring (a) reducing the free radical DPPH, (b) reducing the ABTS^●+^, and (c) reducing Fe^3+^ to Fe^2+^ according to Sánchez-González et al. [37] using standard curves of Trolox. The AA results were expressed in each method in μmol of Trolox equivalents (TE) per mL.

#### Chromatographic Analyses: HPLC and UHPLC

The identification and quantification of sugars, organic acids, phenolic acids, methylxanthines, and flavonoids were performed using chromatographic analysis. An aliquot (1 mL) of each sample was homogenized in 10 mL of water and centrifuged (9056× *g* for 15 min). The supernatant was collected and filtered through a 0.22 µm PVDF filter (Millipore, Cork, Ireland).

For the determination of sugars and organic acids, a Shimadzu LC 20A HPLC (Shimadzu Co., Kyoto, Japan) composed of a pump (LC-20AT), automatic injector (SIL-20AC HT), column oven (CTO-20A) and photodiode array (SPD-M20A) and refractive index (RID-10A) detectors coupled in series (HPLC-PDA-RID) was used. The chromatographic analyses were isocratic. The chromatographic conditions for sugar and organic acid determination are described in the Supplementary Material—Table S1, according to Pauli et al. [38].

The phenolic compounds and methylxanthines were determined according to Terhaag et al. [39]. The results were obtained by analyzing the retention times and areas of the peaks in the chromatograms after comparison with the standard curves of some phenolics and methylxanthines. The results are expressed in μg/mL.

Ethanol content was determined by headspace gas chromatography coupled with mass spectrometry (GC-MS, QP2010-SE-SHIMADZU) equipped with a combi-PAL autosampler (AOC 5000) for liquid and static headspace injection (Shimadzu Co., Kyoto, Japan). The system was automated using 20 mL vials containing 5 mL of either ethanol calibration standards or sample. These were equilibrated at 80 °C for 10 min in headspace mode. The chromatographic conditions employed [40] were Restek Rtx-5MS capillary column (30 m × 0.25 mm; internal diameter of 0.25 μm); helium as a carrier gas with a flow rate of 0.7 mL/min; and column pressure and temperature of 23.3 kPa and 35 °C, respectively. The oven temperature ramp was 35–280 °C, being 1–5 min at a rate of 30 °C/min and from 5–7.5 min at 50 °C/min, and constant temperature was maintained for 7.5–9.43 min. The injector temperature was 150 °C, with detector and interface temperatures of 200 °C and 280 °C, respectively. Volatile compounds were injected with a headspace injection volume of 500 μL at a split ratio of 1:20. The results were obtained by analyzing the retention times and peak areas of the chromatograms and expressed as a percentage of ethanol per the volume of probiotic lychee beverage (*v*/*v*).

#### 2.2.5. Sensory Acceptance

The LB4, LB8, and LB12 beverages on the 1st, 7th, 14th, 21st, and 28th days of cold storage were subjected to acceptance tests. Sensory tests were carried out in individual booths, with procedures approved by the Committee of Ethics in Research Involving Humans (certificate CAAE n° 56478316.8.0000.5231).

To assess acceptance, the team consisted of 53 usual or potential consumers of fruit-based beverages and/or functional beverages (they drank approximately 2.5 L of fruit-based beverages per month). The team consisted of 33 women and 20 men who were younger (76% aged between 18 and 39 years) and had a high level of education (85% undergraduate or graduate).

The evaluators evaluated the color, aroma, flavor, texture, and global acceptability of the beverages through a structured hybrid scale of ten centimeters (0 = dislike extremely; 10 = like extremely) [41].

#### 2.2.6. Statistical Analysis

The experiment was conducted twice in accordance with a completely randomized design (CRD). The physicochemical and microbiological parameters were analyzed weekly for 28 days in triplicate. A split-plot treatment was employed, and sugar content and storage were the main and secondary treatments, respectively. The preparation and storage data were analyzed using ANOVA and Tukey’s test (*p* ≤ 0.05) (Sisvar 5.6 program) [42]. The Pearson test was applied to determine the correlation among probioticated lychee beverage composition, *S. boulardii* viability, and days of cold storage. The significance level of 5% was considered. We employed the software Statistica 7.1 [43].

The sensory analysis data were subjected to two-way ANOVA (for beverages and consumers). Tukey’s test was used to compare means (*p* ≤ 0.05). The Statistica 7.1 program [43] was used in these analyses.

## 3. Results and Discussion

### 3.1. Effect of S. boulardii Probiotic on the Physicochemical Characteristics of Lychee Beverages

The culture viability, TSS, pH, and ethanol content after beverage preparation and fermentation are shown in Table 1. The addition of inoculum, followed by fermentation for 24 h, increased (*p* ≤ 0.05) *S. boulardii* viability in LB (5–6.8 log CFU/mL). Lee and Salminen [44] reported a minimum daily consumption of 6 to 9 log CFU of probiotic microorganisms to obtain some health-beneficial effects. According to some authors, this amount varies depending on the type of probiotic microorganism and the desired health effect [2,3]. In the LB probiotication, the fermentation stage promoted an increase in cell viability, and the beverage became viable enough to be probiotic and promoted a beneficial effect on health.

The probiotication of *S. boulardii* promoted a decrease in TSS because yeasts use sugars as the primary energy source for their metabolism [45]. The fermentation stage was carried out under agitation to provide greater oxygenation in the lychee juice, which contributed to the increase in yeast viability.

During fermentation (Table 1), ethanol (0–4%) and CO_2_ were produced, in addition to a slight reduction in the pH (4.5–3.6) of the LB. This result is similar to those reported by Reitenbach et al. [46] and described by Nelson and Cox [45], who commented that *S. boulardii* metabolizes sugars, mainly producing ethanol and CO_2_ in addition to small amounts of organic acids.

### 3.2. Effect of TSS and Cold Storage on Probiotic Lychee Beverages

#### 3.2.1. *S. boulardii* Viability

Even when stored in refrigerated conditions, LB increased the viability of *S. boulardii* yeast. After the 1st day of cold storage, the CFU/mL ranged from 7.3 to 7.5 log CFU/mL (Table 1). As the lychee juice used to prepare LB contained 4.2, 3.7, and 0.1 g/100 mL fructose, glucose, and sucrose, respectively, the multiplication of yeasts in LB4 occurred due to the natural presence of these fermentable carbohydrates [45,46,47]. In addition to the sugars from the lychee juice, LB8 and LB12 received sucrose supplementation, which increased the yeast viability compared to that of LB4. Gaboardi et al. [48] reported an increase in *S. boulardii* viability after sucrose supplementation in effluents.

All beverages presented greater viability after the 7th day of cold storage. On the 21st day, LB4 and LB8 had viabilities ≥ 5.8 log CFU/mL. On the same day, LB12 showed the greatest increase in viability (6.9 log CFU/mL), decreasing to 5.8 log CFU/mL on the 28th day. Considering that the viability of LB12 was >6.9 log CFU/mL and that this parameter is a prerequisite for its use as a probiotic, LB12 was considered adequate until the 21st day.

#### 3.2.2. Total Soluble Solids (TSS) and pH

**Figure 2:**
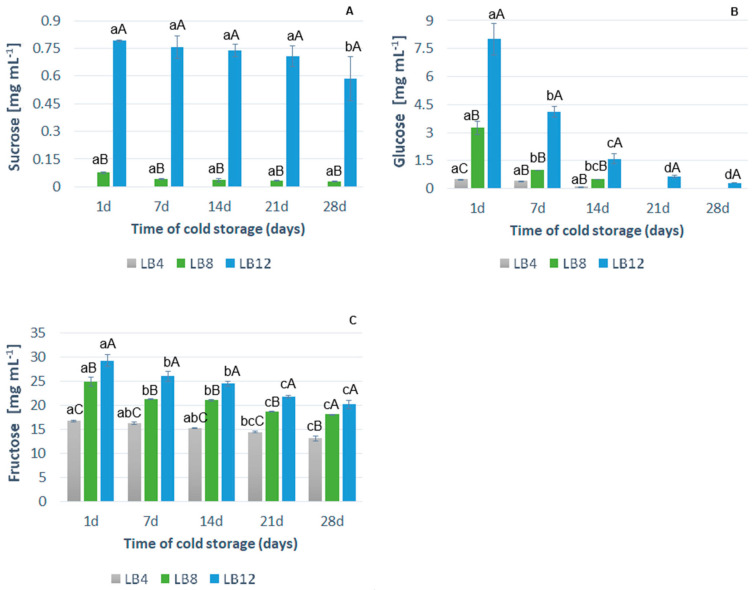
Variation in the content of (**A**) sucrose (mg/mL), (**B**) glucose (mg/mL), and (**C**) fructose (mg/mL) during cold storage of probiotic lychee beverages (LB4, LB8, and LB12). Error bars represent the standard deviation (*n* = 4). Different capital letters indicate significant differences at *p* ≤ 0.05 between each of the formulations for the same storage day. Different lowercase letters indicate significant differences (*p* ≤ 0.05) for the same formulation affected by cold storage time.

Even under refrigeration, *S. boulardii* continued metabolizing mono- and disaccharides as primary carbon sources, increasing cell biomass [45]. In LB4, the TSS concentration remained constant throughout cold storage (Figure 2). LB8 and LB12 showed a gradual decrease in TSS content during cold storage. During cold storage, LB8 decreased the TSS (7.0 to 4.6, 1st to 28th of cold storage, respectively). The same reduction occurred with LB12 (approximately 16% and 45% on the 1st and 28th days, respectively). Greater viability is related to the higher TSS, as proven by a positive and significative Pearson correlation (r = 0.37, *p* ≤ 0.05, Supplementary Material—Table S2).

Regarding the pH, on the 1st day of cold storage, the beverages had different pH values (3.74 (LB4) and 3.59 (LB12)). In LB4, the pH remained constant from the 7th to the 21st day and decreased on the 28th day of storage. The pH in both LB8 and LB12 increased after seven days, followed by a constant decrease until the end of the storage period. The variation in pH occurred concomitantly with the consumption of sugars and the production of organic acids, ethanol, and CO_2_. Cold storage does not promote the synthesis of large amounts of organic acids, as this is not the ideal growth temperature for *S. boulardi* [15,19,45,46], but this amount was sufficient to decay the pH level. Hedin [49] pointed out that there is a positive correlation between the growth of *S. boulardii* and organic acid synthesis, with an inverse correlation with the glucose level. This level of pH decay can be favorable for probiotic lychee beverage conservation and sensory acceptance.

#### 3.2.3. Ethanol Content

Like other yeasts of the genus *Saccharomyces*, *S. boulardii* is a yeast that produces ethanol and CO_2_ from the fermentation of glucose [15,25,45]. Because they contain different TSS (Table 1), LB4, LB8, and LB12 had different ethanol concentrations at the beginning of storage (5.5, 7.5, and 6.2%, respectively). The ethanol content remained constant throughout cold storage in LB4 and LB8. Factors such as the composition of the medium (mainly the TSS level) and the accumulation of ethanol interfere with the viability and fermentative capacity of the yeast [45,48], which explains the ethanol constancy level of LB4 and LB8. *S. boulardii* maintains viability even when ethanol is present [15,25], allowing mildly alcoholic beverages to take on probiotic characteristics.

For LB12, there was an increase in the ethanol content of 40% and 63% at the 21st and 28th days of storage, respectively, due to the continuity of yeast metabolism, even during cold storage. On the 28th day of cold storage, LB12 contained 10.1% ethanol, probably due to the metabolism of the remaining sugars and their conversion to alcohol. Cold storage does not prevent ethanol synthesis in these beverages. There was a significant and positive correlation (r = 0.69, *p* ≤ 0.05, Supplementary Material—Table S2) between the SST before fermentation and the alcohol content of the beverages and between the storage time and the alcohol content (r = 0.45, *p* ≤ 0.05).

Considering that moderate alcohol consumption may not necessarily pose health risks [50], and given the need for individualized assessment, the probiotication of lychee beverage represents a viable approach to enhance its nutritional profile, combining pleasure and health benefits. Mena and Aryana [51] even suggest that yogurt containing ethanol and fermented by *L. bulgaricus* and *S. thermophilus* may have a therapeutic effect.

#### 3.2.4. Sugars Content

There were no detectable sucrose levels in LB4 (Figure 2A) and a decrease in glucose (Figure 2B) during cold storage, demonstrating that these nutrients had already been consumed by the yeast during fermentation. On the 1st day of cold storage, 0.08 and 0.78 mg/mL of sucrose were detected in LB8 and LB12, respectively (Figure 2A). There were decreases of approximately 63% and 27%, respectively, until the 28th day. Similarly, for LB8 and LB12, there was a decrease in glucose content (Figure 2B), with a maximum on the 1st day and a minimum on the 14th day. After this period, glucose was not detected. At lower glucose levels, *S. boulardii* preferred sucrose to other sugars. Gaboardi et al. [48] reported similar results in rice effluent when observing a preference for *S. boulardii* to use sucrose over other organic matter.

Among the sugars detected and quantified, fructose was the only one that remained present in the LB4 beverage until the end of cold storage (Figure 2C). At the start of cold storage, LB8 and LB12 presented 24.9 and 29.3 mg/mL of fructose, representing decreases of 27.5% and 31.1%, respectively, throughout the storage period. Thus, *S. boulardii* did not prefer to metabolize fructose in the probiotication of the lychee beverage since this sugar remained detrimental to the decay of other sugars.

Sulieman et al. [52] verified similar results in sugary date palm syrups fermented by *S. cerevisiae*. These authors reported a significant reduction in sucrose and glucose content and maintenance of fructose in the syrup fermented for 72 h. This type of metabolic preference is typical of the *Saccharomyces* genus [45,48].

The presence of fructose in probioticated LB can be desirable for sensory and nutritional reasons since this monosaccharide has a relative sweetness of 1.3 relative to that of sucrose [53]. Fructose can increase sweetness and make beverages more accepted by consumers, in addition to providing a lower caloric value per gram.

#### 3.2.5. Organic Acid Content

Some acids identified in LB are malic, acetic, and citric acids. These organic acids naturally occur in fruit and are also derived from substances involved in the glycolysis of yeast cells [11,45].

**Figure 3:**
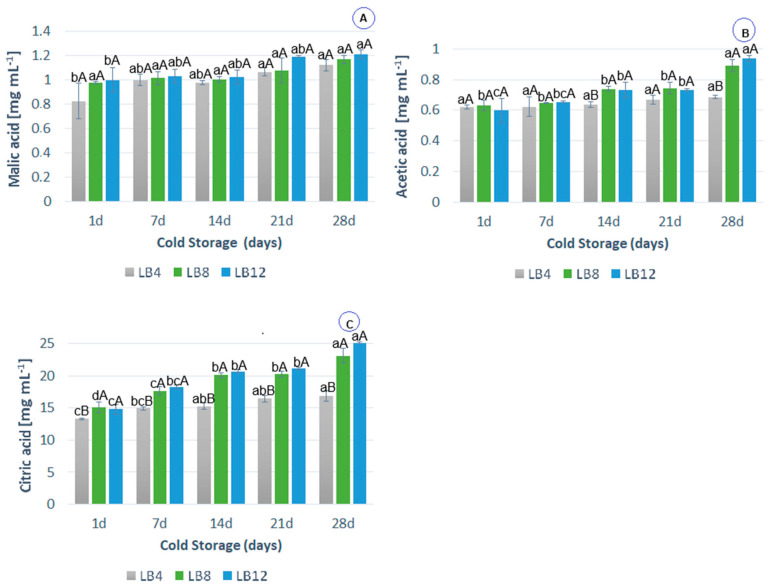
Variation in the content of (**A**) malic acid (mg/mL), (**B**) acetic acid (mg/mL), and (**C**) citric acid (mg/mL) in cold-stored probiotic lychee beverages (LB4, LB8, and LB12). Error bars represent the standard deviation (*n* = 4). Different capital letters indicate significant differences at *p* ≤ 0.05 between each of the formulations for the same storage day. Different lowercase letters indicate significant differences (*p* ≤ 0.05) for the same formulation affected by cold storage time.

During cold storage, there was an increase in the organic acid content (Figure 3A–C) in the probiotic lychee beverages. The acetic acid content (Figure 3B) in LB4 remained constant, with increases in LB8 and LB12 (41% and 56%, respectively) at the end of cold storage. There was an increase of 27% (LB4), 52% (LB8), and 69% (LB12) in citric acid during storage (Figure 3C). The addition of probiotic lychee beverages containing more sucrose resulted in a higher organic acid content. This result is expected because *S. boulardii* produces organic acids through the metabolism of sugars during anaerobic fermentation.

#### 3.2.6. Total Phenolic Compounds (TPCs) and Antioxidant Activity (AA)

On the 1st day of cold storage, the TPC levels in the probiotic lychee beverages were 151.7, 133.7, and 190.1 µg GAE/mL (LB4, LB8, and LB12, respectively). Similar results were reported by Değirmencioğlu et al. [14] for radish juice fermented by *S. boulardii* and other fungi. In general, there was a decrease in the TPC and AA content in LB during cold storage (Table 2).

Despite LB12 having a greater TPC than the other beverages, at the end of cold storage, all beverages had similar phenolic contents. Some factors, such as nutrient levels, fermentation conduction, exposure to oxygen and light, and intermolecular interactions, can interfere with the TPC content and (probably) the AA content [47,54,55]. There was a reduction in TPC during cold storage of approximately 20% (LB4), 8% (LB8), and 26% (LB12). The same reduction in the TPC of juices probioticated by *S. boulardii* during cold storage was observed in berry juice (7.8%) [5] and tomato juice (3.6%) [21].

The AA content measured by the DPPH assay decreased during cold storage (57.5%, 68.8%, and 41.2% for LB4, LB8, and LB12, respectively). Additionally, a decrease in AA was detected using the FRAP and ABTS methods. The AA content of the probiotic beverages was similar according to the DPPH and FRAP assays at the end of cold storage. Only the ABTS assay showed a significant decrease in AA.

There was also a significant (*p* ≤ 0.05) positive correlation between TPC and AA according to the DPPH, FRAP, and ABTS assays (r = 0.39; r = 0.66; r = 59, respectively, Supplementary Material—Table S2). Therefore, most of the phenolic compounds in LB are related to its antioxidant activity.

#### 3.2.7. Phenolic Acid, Flavonoid and Methylxanthine Contents

Through chromatographic analysis, 21 bioactive compounds were separated from probiotic lychee beverages. The following were identified in ascending order of retention time: trigonelline, ascorbic acid, nicotinic acid, gallic acid, protocatechuic acid, theobromine, paraxanthine, theophylline, epigallocatechin, catechin, chlorogenic acid, caffeic acid, caffeine, epicatechin, p-coumaric acid, ferulic acid, sinapic acid, rutin, myricetin, quercetin, and kaempferol.

**Figure 4:**
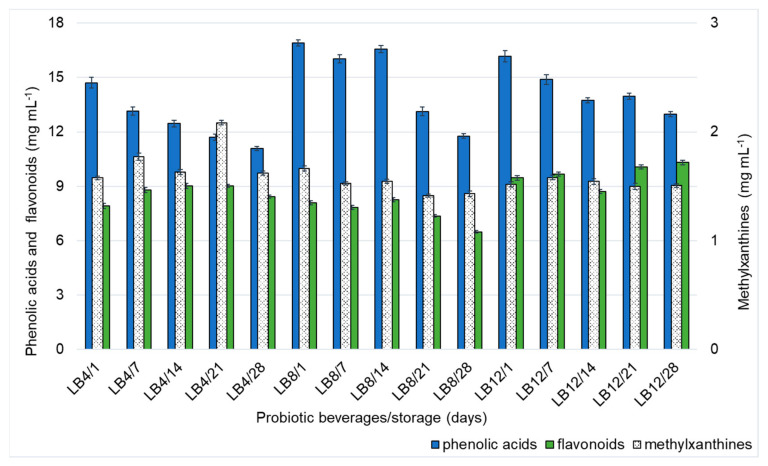
Variation in phenolic acids, flavonoids, and methylxanthines in probiotic lychee beverages (LB4, LB8, and LB12) during cold storage. Error bars represent the standard deviation (*n* = 4). LB4 (probiotic lychee beverage), LB8 (probiotic lychee beverage adjusted to 8 °Brix), and LB12 (probiotic lychee beverage adjusted to 12 °Brix).

Except for trigonelline (9.36, 9.26, and 8.56 mg/mL in LB4, LB8, and LB12, respectively), ascorbic acid (213.1, 95.1, and 110.7 mg/mL, in LB4, LB8 and LB12, respectively), and nicotinic acid (0.24, 0.25, 0.18 mg/mL, in LB4, LB8 and LB12, respectively), the compounds were grouped into three classes—phenolic acids, flavonoids, and methylxanthines—to facilitate the discussion of the results. The concentrations belonging to each class were summed (Figure 4). The compounds classified as phenolic acids are gallic acid, protocatechuic acid, chlorogenic acid, caffeic acid, p-coumaric acid, ferulic acid, and sinapic acid. The flavonoids were epigallocatechin, catechin, epicatechin, rutin, myricetin, quercetin, and kaempferol. The methylxanthines used were caffeine, theobromine, paraxanthine, and theophylline.

In LB4, there was a decrease in phenolic acid content and a slight increase in methylxanthine and flavonoid content during storage. The levels of phenolic acids, flavonoids, and methylxanthines decreased in LB8 during storage (30%, 14%, and 19%, respectively). For LB12, there was an increase in the flavonoid content of approximately 9% over the 28 days of storage and, on the other hand, a 19% decrease in the phenolic acid content and maintenance of the methylxanthine content.

Research indicates that the action of microorganisms during fermentation—metabolizing the substrate and providing molecular interactions, including with the oxygen present in the medium—can interfere with the profile and content of phenolic acids, methylxanthines, and flavonoids [14,54,55]. Thus, the different sucrose contents of the probiotic lychee beverages differed during storage in terms of yeast viability, ethanol content, sugar content, organic acid content, and phenolic compound content.

Another aspect that should be mentioned is that the presence of phenolic acids and flavonoids can help in the positive modulation of the intestinal microbiota, even in ethanol. Queipo-Ortuño et al. [30] reported in research that red wines (alcoholic or non-alcoholic) promote microbiota selection in addition to improving metabolic parameters in humans. They suggest that despite containing ethanol, the beverage can have a beneficial effect if it presents high levels of bioactivity. Thus, the probiotication of lychee beverage, rich in phenolic acids and flavonoids, even containing ethanol, can be an alternative to the probiotic beverages currently on the market.

### 3.3. Sensory Acceptability of Probiotic Lychee Beverages

The probiotic lychee beverage LH12 was chosen for sensory acceptability because it showed greater viability of *S. boulardii* and higher TPC and TSS at the end of the cold storage period. To meet the requirement of being probiotic, LB12 samples whose viability was <6 log CFU/mL on the date of sample collection were excluded. Due to the viability > 6 log CFU/mL up to the 21st day, LB12 acceptance on the 1st, 7th, 14th, and 21st days of cold storage was determined. These beverages were renamed LB12/1, LB12/7, LB12/14, and LB12/21 (collected on the 1st, 7th, 14th, and 21st days of cold storage, respectively). Table 3 shows the sensory acceptance of the probiotic lychee beverages LB12/1, LB12/7, LB12/14, and LB12/21 in terms of color, aroma, flavor, texture attributes, and global acceptability.

In general, all beverages were sensorially accepted (mean scores > 6.6, Table 3). The beverages had similar (*p* ≤ 0.05) aromas, flavors, textures, and overall acceptability. LB12/14 presented a similar color to that of LB12/1, and both had better colors than LB12/7 and LB12/21. These results indicate that the physicochemical characteristics of LB12 differed during storage but did not affect consumer preference.

The consumers highlighted several characteristics throughout the sensory evaluations: (a) the appreciation of the texture due to the presence of bubbles from the CO_2_ produced by the action of *S. boulardii* and (b) the aroma and global acceptance positively related to the “attractive aroma” and aroma “very similar to a sparkling wine”. These reports point to the positive effect of lychee juice probiotication by *S. boulardii*, as it can make it more sensitive to products already accepted and known by consumers. Mullero-Cerezo et al. [56] also reported a good acceptance of craft beer produced by *S. boulardii*, suggesting its potential use in this beverage. Similarly, these authors reported good acceptability in rose wine [17] fermented by *S. boulardii*.

Conversely, Heenan et al. [24] noted that sensory consumers reported the presence of undesirable flavors in probiotic soy ice cream supplemented with *S. boulardii*. Lourens-Hattingh and Viljoen [22] pointed out that *S. boulardii* is unfeasible as a probiotic agent in flavored yogurts and fruit yogurts because it causes the formation of excessive amounts of ethanol and CO_2_, which are considered undesirable by the sensory evaluators of this product.

## 4. Conclusions

The probiotication of lychee beverages by *S. boulardii* promotes a suitable health-beverage alternative to consumers. When sucrose is added to the probiotic lychee beverage, it promotes acceptable physicochemical and sensory characteristics, in addition to complying with the longest possible cold storage time, with the condition of being a probiotic beverage. Further studies should be conducted to verify the in vivo effect of ingesting the probiotic lychee beverage.

## Figures and Tables

**Table 1:** Physicochemical and microbiological parameters of probiotic lychee beverage ^1^.

Parameters	Step	Time	Formulations ^2^		
			LB4	LB8	LB12
Viability (log CFU/mL)	Preparation ^3^	0 h	5.0 ± 0.0 ^dA^	5.0 ± 0.0 ^eA^	5.0 ± 0.0 ^fA^
		24 h	6.8 ± 0.0 ^cA^	6.8 ± 0.0 ^cdA^	6.8 ± 0.0 ^deA^
	Cold storage	1 d	7.4 ± 3.6 ^bB^	7.3 ± 3.4 ^bB^	7.5 ± 2.7 ^cA^
		7 d	7.6 ± 2.9 ^aC^	7.7 ± 1.0 ^aB^	7.8 ± 3.6 ^aA^
		14 d	6.3 ± 0.9 ^cB^	6.9 ± 0.8 ^cB^	7.8 ± 3.5 ^bA^
		21 d	5.9 ± 0.1 ^cB^	5.8 ± 0.1 ^cdB^	6.9 ± 0.5 ^dA^
		28 d	5.7 ± 0.3 ^cA^	5.7 ± 0.0 ^cdA^	5.8 ± 0.0 ^eA^
TSS (°Brix)	Preparation ^3^	0 h	12.0 ± 0.0 ^aA^	12.0 ± 0.0 ^aA^	12.0 ± 0.0 ^aA^
		24 h	4.1 ± 0.0 ^bA^	4.1 ± 0.0 ^fA^	4.1 ± 0.0 ^bA^
	Cold storage	1 d	3.7 ± 0.1 ^cC^	7.0 ± 0.1 ^bB^	10.1 ± 0.0 ^bA^
		7 d	3.7 ± 0.0 ^cC^	5.9 ± 0.0 ^bB^	9.5 ± 0.0 ^cA^
		14 d	3.7 ± 0.0 ^cC^	5.1 ± 0.0 ^cB^	8.5 ± 0.1 ^dA^
		21 d	3.7 ± 0.0 ^cC^	4.7 ± 0.0 ^dB^	7.6 ± 0.1 ^eA^
		28 d	3.7 ± 0.0 ^cC^	4.6 ± 0.1 ^eB^	6.6 ± 0.4 ^fA^
pH	Preparation ^3^	0 h	4.5 ± 0.0 ^aA^	4.5 ± 0.0 ^aA^	4.5 ± 0.0 ^aA^
		24 h	3.6 ± 0.0 ^bcA^	3.8 ± 0.0 ^dA^	3.8 ± 0.0 ^cA^
	Cold storage	1 d	3.7 ± 0.0 ^cA^	3.7 ± 0.1 ^eB^	3.6 ± 0.0 ^eC^
		7 d	3.8 ± 0.0 ^bB^	3.9 ± 0.0 ^bA^	3.8 ± 0.0 ^bB^
		14 d	3.8 ± 0.0 ^bcB^	3.8 ± 0.0 ^cA^	3.7 ± 0.0 ^dC^
		21 d	3.8 ± 0.0 ^bcA^	3.8 ± 0.0 ^dA^	3.6 ± 0.0 ^dC^
		28 d	3.4 ± 0.0 ^dB^	3.5 ± 0.1 ^fA^	3.5 ± 0.0 ^fA^
Ethanol (%)	Preparation ^3^	0 h	0.0 ± 0.0 ^cA^	0.0 ± 0.0 ^cA^	0.0 ± 0.0 ^fA^
		24 h	4.0 ± 0.1 ^bA^	4.0 ± 0.1 ^bA^	4.0 ± 0.1 ^eA^
	Cold storage	1 d	5.6 ± 0.1 ^aB^	7.5 ± 0.29 ^aA^	6.2 ± 0.2 ^dB^
		7 d	5.6 ± 0.1 ^aB^	7.5 ± 0.02 ^aA^	7.1 ± 0.0 ^cA^
		14 d	5.8 ± 0.0 ^aB^	7.8 ± 0.77 ^aA^	7.8 ± 0.1 ^cA^
		21 d	6.1 ± 0.1 ^aB^	8.1 ± 0.03 ^aA^	8.7 ± 0.0 ^bA^
		28 d	6.2 ± 0.2 ^aC^	8.1 ± 0.06 ^aB^	10.1 ± 0.9 ^aA^

^1^ Results are expressed as the mean (*n* = 6) ± standard deviation. Different capital letters on the same line indicate significant differences (*p* ≤ 0.05) between each of the formulations for the same day of cold storage. Different lowercase letters in the same column and for the same characteristic indicate significant differences (*p* ≤ 0.05) in the formulation affected by storage time. ^2^ Formulation: LB4 (probiotic lychee beverages), LB8 (probiotic lychee beverages adjusted to 8 °Brix), and LB12 (probiotic lychee beverages adjusted to 12 °Brix). ^3^ Preparation: this step included fermentation for 24 h at 30 °C under agitation and TSS adjustment.

**Table 2:** Variation in the TPC and AA content of probiotic lychee beverages during cold storage ^1^.

Parameters	Time of Cold Storage (Days)	Formulations ^2^		
		LB4	LB8	LB12
TPC ^3^	1	151.67 ± 7.32 ^Ab^	133.70 ± 6.67 ^bcB^	190.08 ± 4.30 ^abA^
	7	144.46 ± 3.09 ^abB^	157.01 ± 2.38 ^abB^	202.10 ± 34.00 ^Aa^
	14	129.34 ± 4.56 ^abB^	169.49 ± 2.65 ^aA^	176.68 ± 5.89 ^bcA^
	21	121.87 ± 1.17 ^bB^	123.55 ± 3.28 ^bC^	153.17 ± 1.46 ^cdA^
	28	121.87 ± 1.97 ^aB^	122.80 ± 2.44 ^aC^	140.13 ± 0.84 ^aD^
DPPH ^4^	1	0.73 ± 0.04 ^Aa^	0.80 ± 0.11 ^aA^	0.51 ± 0.15 ^abB^
	7	0.59 ± 0.09 ^abA^	0.69 ± 0.20 ^aA^	0.68 ± 0.13 ^aA^
	14	0.43 ± 0.13 ^bcAB^	0.64 ± 0.04 ^aA^	0.41 ± 0.11 ^bB^
	21	0.33 ± 0.02 ^aC^	0.27 ± 0.01 ^aB^	0.33 ± 0.01 ^aB^
	28	0.31 ± 0.02 ^aC^	0.25 ± 0.01 ^aB^	0.30 ± 0.03 ^aB^
FRAP ^4^	1	1.20 ± 0.07 ^aA^	1.19 ± 0.02 ^Aa^	1.13 ± 0.02 ^aB^
	7	0.82 ± 0.06 ^bcB^	1.24 ± 0.10 ^Aa^	1.23 ± 0.03 ^aA^
	14	0.90 ± 0.02 ^cB^	1.00 ± 0.02 ^Bb^	1.18 ± 0.02 ^abA^
	21	0.79 ± 0.05 ^abC^	0.73 ± 0.01 ^Cb^	0.85 ± 0.01 ^aC^
	28	0.78 ± 0.01 ^aC^	0.78 ± 0.02 ^Ca^	0.71 ± 0.04 ^aD^
ABTS ^4^	1	6.11 ± 0.33 ^aA^	5.42 ± 0.71 ^bB^	5.61 ± 0.13 ^abB^
	7	4.23 ± 0.02 ^bB^	6.29 ± 0.16 ^aA^	6.29 ± 0.16 ^aA^
	14	2.44 ± 0.15 ^bC^	3.32 ± 0.05 ^aC^	3.30 ± 0.10 ^aC^
	21	1.71 ± 0.15 ^aD^	0.29 ± 0.06 ^bD^	0.28 ± 0.08 ^bD^
	28	0.87 ± 0.05 ^aB^	0.11 ± 0.05 ^bD^	0.03 ± 0.00 ^bD^

^1^ Results are expressed as the mean (*n* = 6) ± standard deviation. Different capital letters on the same line indicate significant differences (*p* ≤ 0.05) between each of the formulations for the same day of cold storage. Different lowercase letters in the same column and for the same characteristic indicate significant differences (*p* ≤ 0.05) in the formulation affected by storage time. ^2^ LB4 (probiotic lychee beverages), LB8 (probiotic lychee beverages adjusted to 8 °Brix), and LB12 (probiotic lychee beverages adjusted to 12 °Brix). ^3^ Results are presented as gallic acid equivalents per mL of beverage (µg GAE/mL). ^4^ Results are reported as Trolox equivalents per mL of beverage (µmol TEAC/mL).

**Table 3:** Acceptability of probiotic lychee beverage ^1^.

	Parameters ^3^				
Formulations ^2^	Color	Aroma	Flavor	Texture	Global Acceptability
LB12/1	7.5 ± 1.5 ^ab^	7.8 ± 1.6 ^a^	7.7 ± 1.9 ^a^	7.9 ± 1.6 ^a^	7.7 ± 1.7 ^a^
LB12/7	6.6 ± 2.2 ^c^	7.6 ± 1.8 ^a^	7.4 ± 1.9 ^a^	7.8 ± 1.6 ^a^	7.4 ± 1.7 ^a^
LB12/14	7.8 ± 1.4 ^a^	7.9 ± 1.4 ^a^	7.5 ± 1.8 ^a^	8.0 ± 1.5 ^a^	7.7 ± 1.5 ^a^
LB12/21	6.9 ± 2.1 ^bc^	7.7 ± 1.4 ^a^	7.6 ± 1.8 ^a^	7.9 ± 1.2 ^a^	7.5 ± 1.6 ^a^

^1^ Results ± standard deviation. Different letters for the same attribute represent a significant difference (*p* ≤ 0,05). ^2^ LB12/1 (probiotic lychee beverage adjusted to 12 °Brix, cold stored for 1 day); LB12/7 (probiotic lychee beverage adjusted to 12 °Brix, cold stored for 7 days); LB12/14 (probiotic lychee beverage adjusted to 12 °Brix, cold stored for 14 days); LB12/21 (probiotic lychee beverage adjusted to 12 °Brix, cold stored for 21 days). ^3^ Hedonic values (color, aroma, flavor, texture, and global acceptability): 0 = disliked very much; 10 = I liked it very much.

## Data Availability

The original contributions presented in the study are included in the article/Supplementary Material, further inquiries can be directed to the corresponding author.

## Supplementary Materials

The following supporting information can be downloaded at: https://www.mdpi.com/article/10.3390/foods14020156/s1, Table S1: Chromatographic conditions used for the determination of sugars and organic acids, Table S2: Pearson correlation coefficients between probioticated lychee beverage composition, *S. boulardii* viability and days of cold storage.
