# Supplementary material for: The Probiotication of a Lychee Beverage with Saccharomyces boulardii: An Alternative to Dairy-Based Probiotic Products
Source: Foods. 2025 Jan 7;14(2):156. doi: 10.3390/foods14020156 (PMC11764993; doi:10.3390/foods14020156)
Supplement: Supplementary file 1 [file foods-14-00156-s001.zip › foods-3313428-supplementary.pdf]

Supplementary material

Supplementary Material:Table S1 - Chromatographic conditions used for the determination of sugars and organic acids [1]

	Sugars	Organic Acids
Mobile phase	100% Ultrapure water	100% Phosphate buffer 25mM pH 2.4
Column	Aminex HPX-87P (7.8 x 300 mm; Biorad, CA, USA)	CapCell Pak C18 (250 x 4.6mm x 5µm; Shiseido Co. Ltd., Japan)
Oven temperature (°C)	85	40
Detector temperature (°C)	40	40
Flow rate (mL min^{-1})	1.0	1.0
Injection volume (µL)	20	20

According to Pauli et al (2011)

Supplementary Material: Table S2 - Pearson correlation coefficients between probioticated lychee beverage composition, S. *boulardii* viability and days of cold storage

	**Cold storage**	**TSS initial**	**Yeast viability**	**TSS**	**TPC**	**DPPH**	**FRAP**	**ABTS**	**EtOH**
**Cold storage**	1.00	0.00	-0.63	-0.32	-0.52	-0.81	-0.81	-0.93	0.45
**TSS initial**	0.00	1.00	0.37	0.90	0.60	-0.06	0.25	0.01	0.69
**Yeast viability**			1.00	0.58	0.73	0.55	0.77	0.76	-0.12
**TSS**				1.00	0.75	0.17	0.51	0.33	0.35
**TPC**					1.00	0.39	0.66	0.59	0.02
**DPPH**						1.00	0.72	0.84	-0.29
**FRAP**							1.00	0.88	-0.19
**ABTS**								1.00	-0.44
**EtOH**									1.00

*Significant terms ($p \leq 0.05$);

Cold storage: days of cold storage (1 to 28 d). TSS initial: total soluble solids (°Brix) after preparation (t=0). Yeast viability log CFU/mL of beverage. TSS: total soluble solids (°Brix). TPC: Total phenolic content (µg EAG mL^{-1}). DPPH: antioxidant activity by DPPH (µmol TEAC mL^{-1}). FRAP: antioxidant activity by FRAP (µmol TEAC mL^{-1}). ABTS: antioxidant activity by ABTS (µmol TEAC mL^{-1}. EtOH: ethanol % (v/v).
